# Economic evaluations of human schistosomiasis interventions: a systematic review and identification of associated research needs

**DOI:** 10.12688/wellcomeopenres.15754.2

**Published:** 2020-08-07

**Authors:** Hugo C. Turner, Michael D. French, Antonio Montresor, Charles H. King, David Rollinson, Jaspreet Toor

**Affiliations:** 1 MRC Centre for Global Infectious Disease Analysis, Department of Infectious Disease Epidemiology, School of Public Health, Faculty of Medicine, St Mary’s Campus, Imperial College London, London, W2 1PG, UK; 2 Oxford University Clinical Research Unit, Wellcome Africa Asia Programme, Ho Chi Minh City, Vietnam; 3 Centre for Tropical Medicine and Global Health, Nuffield Department of Medicine, University of Oxford, Oxford, UK; 4 RTI International, Washington, 20005, USA; 5 Control of Neglected Tropical Diseases, World Health Organization, Geneva, Switzerland; 6 Center for Global Health and Diseases, Case Western Reserve University, Cleveland, USA; 7 Global Schistosomiasis Alliance, Natural History Museum, London, UK; 8 Big Data Institute, Li Ka Shing Centre for Health Information and Discovery, University of Oxford, Oxford, UK

**Keywords:** Schistosomiasis, NTDs, Economic evaluations, Cost-benefit, Cost-effectiveness, Cost per DALY averted, Preventive chemotherapy, MDA

## Abstract

**Background: **Schistosomiasis is one of the most prevalent neglected tropical diseases (NTDs) **with an estimated 229 million people requiring preventive treatment worldwide. Recommendations for preventive chemotherapy strategies have been made by the World Health Organization (WHO) whereby the frequency of treatment is determined by the settings prevalence. Despite recent progress, many countries still need to scale up treatment and important questions remain regarding optimal **control strategies. This paper presents a systematic review of the economic evaluations of human schistosomiasis interventions.

**Methods: **A systematic review of the literature was conducted on 22nd August 2019 using the PubMed (MEDLINE) and ISI Web of Science electronic databases. The focus was economic evaluations of schistosomiasis interventions, such as cost-effectiveness and cost-benefit analyses. No date or language stipulations were applied to the searches.

**Results: **We identified 53 relevant health economic analyses of schistosomiasis interventions. Most studies related to *Schistosoma japonicum* followed by *S. haematobium. *Several studies also included other NTDs. In Africa, most studies evaluated preventive chemotherapy, whereas in China they mostly evaluated programmes using a combination of interventions (such as chemotherapy, snail control and health education). There was wide variation in the methodology and epidemiological settings investigated. A range of effectiveness metrics were used by the different studies.

**Conclusions: **Due to the variation across the identified studies, it was not possible to make definitive policy recommendations. Although, in general, the current WHO recommended preventive chemotherapy approach to control schistosomiasis was found to be cost-effective. This finding has important implications for policymakers, advocacy groups and potential funders. However, there are several important inconsistencies and research gaps (such as how the health benefits of interventions are quantified) that need to be addressed to identify the resources required to achieve schistosomiasis control and elimination.

## Introduction

Schistosomiasis (also known as bilharzia) is an acute and chronic parasitic disease caused by blood flukes of the genus *Schistosoma*. People become infected when larval forms of the parasite (released by freshwater snails) penetrate the skin during contact with infested water. It is one of the most prevalent neglected tropical diseases (NTDs) with an estimated 229 million people requiring preventive treatment worldwide ^1^. There are two main forms of human schistosomiasis: urogenital caused by *S. haematobium,* and intestinal caused by *S. mansoni, S. guineensis, S. intercalatum, S. japonicum,* and *S. mekongi.* Schistosomiasis can result in anaemia, chronic pain, diarrhea, and malnutrition, causing poor school performance and lower fitness ^2^. It is estimated that 89.3% of those requiring treatment for schistosomiasis live in Africa ^1^.

In Africa, schistosomiasis control mainly focuses on mass school-based or community-wide preventive chemotherapy using praziquantel ^3^: the large-scale distribution of drugs to eligible populations, without diagnosing or testing individuals for current infection. The World Health Organization’s (WHO) recommended guidelines for preventive chemotherapy are currently dependent on the prevalence of infection in school-aged children (SAC; 5–14 years old) prior to preventive chemotherapy ^4^. Although SAC are generally the focus using targeted school-based preventive chemotherapy, treatment of adults is also recommended depending on the endemicity. Specifically, in low-risk communities (below 10% baseline prevalence in SAC), treatment of SAC once every 3 years is recommended, along with treatment of suspected cases ^4^. For moderate-risk communities (10–50% baseline prevalence in SAC), biennial treatment of SAC and at-risk adults is recommended ^4^. For high-risk communities (>50% baseline prevalence in SAC), annual treatment of SAC and at-risk adults is recommended ^4^. Outside of Africa, it is more common for preventive chemotherapy to be complemented by other interventions, such as health education and snail control ^5– 8^.

Merck KGaA have committed to donate 250 million tablets of praziquantel annually for the treatment of schistosomiasis, primarily for SAC in Africa ^9^. The current WHO goals of morbidity control and elimination as a public health problem ^10^, focus on preventive chemotherapy to reduce the prevalence of heavy-intensity infections in SAC and furthermore the interruption of transmission in selected settings (reducing the incidence of infections to zero) ^11, 12^ ( Box 1). The post-2020 WHO goals are currently being developed, along with revisions to the current preventive chemotherapy guidelines.

Box 1. **Glossary**

**Cost-benefit analysis:** A type of economic evaluation in which both the costs and the resulting outcomes (i.e. health benefits) of the interventions in question, are expressed in monetary terms. The results are typically expressed by a benefit-cost ratio.

**Cost-effectiveness analysis:** A type of economic evaluation in which the cost of the interventions in question are compared to the quantity of a non-monetary effectiveness measure (such as the number of deaths or cases averted). This avoids the challenges associated with monetising the benefits of healthcare interventions. The results are expressed as a cost per unit of outcome (see cost-effectiveness ratio).

**Cost-effectiveness ratio:** A statistic used to summarise the cost-effectiveness of an intervention. It is calculated by dividing the cost of an intervention by its effectiveness measure, such as a cost per disability-adjusted life year (DALY) averted. An **incremental cost-effectiveness ratio** is calculated by dividing the difference in costs by the difference in effectiveness outcomes of two alternative options (it summarises the ‘extra cost per additional unit of effect gained’).

**Cost-utility analysis:** A specific subtype of cost-effectiveness analysis, where the effectiveness of the intervention is measured using a "utility-based" unit (such as disability-adjusted life years (DALYs) averted and quality-adjusted life years (QALYs) gained).

**Disability-adjusted life years (DALYs):** A measure of disease burden that is calculated as the sum of the years of life lost due to premature mortality and the years of healthy life lost due to disability. The number of years of healthy life lost due to disability is calculated using a disability weight factor between 0 and 1, that reflects the severity of the disease/disability (with 0 representing perfect health and 1 representing death). One DALY can be thought of as one year of "healthy" life lost.

**Economic costs:** These define the cost of a resource as its value in its next best alternative use that has been forgone (also known as an opportunity cost). This is a broader conceptualization of a resource’s value than its financial cost, as it recognizes that using a resource makes it unavailable for productive use elsewhere. The rationale behind economic costs is that they are intended to represent the full value of the resources used for an intervention, and they account for the fact that resources can have a value that is not (fully) captured by their financial costs (such as the ‘free’ use of building space provided by Ministries of Health, and the unpaid time devoted to mass preventive chemotherapy programmes by volunteers). This is particularly important when considering issues related to the sustainability and replicability of interventions.

**Economic evaluation:** Economic evaluations are a specific type of health economic analysis that formally evaluates the costs and benefits of two (or more) alternative courses of action.

**Elimination as a public health problem:** Goal defined by achieving <1% prevalence of heavy-intensity infections in school-aged children ^10^.

**Financial costs:** The actual expenditure (i.e. the amount paid) for the goods, resources and services that are purchased.

**Heavy-intensity infections:** For intestinal schistosomiasis caused by *S. mansoni*, heavy-intensity infections are defined as greater than 400 eggs per gram of stool and for urogenital schistosomiasis caused by *S. haematobium*, this is defined as over 50 eggs per 10 mL of urine ^10^.

**Interruption of transmission (breaking transmission):** End goal defined by reducing the incidence of infections to zero ^10^.

**Morbidity control:** Goal defined by achieving <5% prevalence of heavy-intensity infections in school-aged children ^10^.

**Prevalence settings:** Defined as low with below 10% baseline (prior to treatment) prevalence in school-aged children; moderate with 10–50% baseline prevalence in school-aged children; high with above 50% baseline prevalence in school-aged children ^4, 10^.

**Time horizon:** The time horizon of an economic evaluation determines the duration over which the outcomes (i.e. the effectiveness) and costs are calculated.

**Mass preventive chemotherapy:** The large-scale distribution of drugs to eligible populations, without diagnosing or testing individuals for current infection ^3^.

**Quality-adjusted life year (QALY):** A outcome measure used to quantify the effectiveness of a particular intervention. QALYs have been designed to capture both gains in quality and quantity of life. The utility weights used to estimate the gains in quality of life are measured on a scale where perfect health is valued as 1 and death as 0.

The treatment coverage of SAC has increased notably over the last decade, reaching 61.2% in 2018 ^1^. However, despite the WHO recommendations, adults are often missed, and their coverage is markedly lower (18.2%) ^1^. Although most treatment programmes currently rely solely on preventive chemotherapy, additional operational components, such as the provision of potable water and adequate sanitation, hygiene education, behaviour change and snail control may become essential when moving towards elimination of schistosomiasis ^13^.

Despite recent progress, important questions remain regarding optimal schistosomiasis strategies ^14^. Economic evaluations have an important role in informing strategies and policy decisions. The aim of this paper is to provide a descriptive overview of the economic evaluations that have been conducted, the areas of research focus and the key findings for human schistosomiasis interventions. Based on these findings, we describe important areas of uncertainty, drivers of variation and remaining research gaps that require further attention within future economic evaluations for schistosomiasis.

## Methods

A systematic review of the literature was conducted on 22 ^nd^ August 2019 using the PubMed (MEDLINE) and ISI Web of Science electronic databases. The focus was economic evaluations of schistosomiasis interventions, such as cost-effectiveness and cost-benefit analyses. Although not technically an economic evaluation, studies reporting estimated economic benefits of schistosomiasis interventions were also included. Variants of the following search terms were used to find relevant papers: schistosomiasis and either cost-benefit, cost-effectiveness, economic(s), or economic evaluation. No specific date or language stipulations were applied to the searches. A more detailed summary of the search terms and the PRISMA checklist are supplied as extended data. Studies on schistosomiasis interventions that were identified as a relevant type of economic analysis were included. Studies related to health economic evaluation of schistosomiasis diagnostics/monitoring, cost of illness and willingness to pay for treatment were excluded.

**Figure 1.**
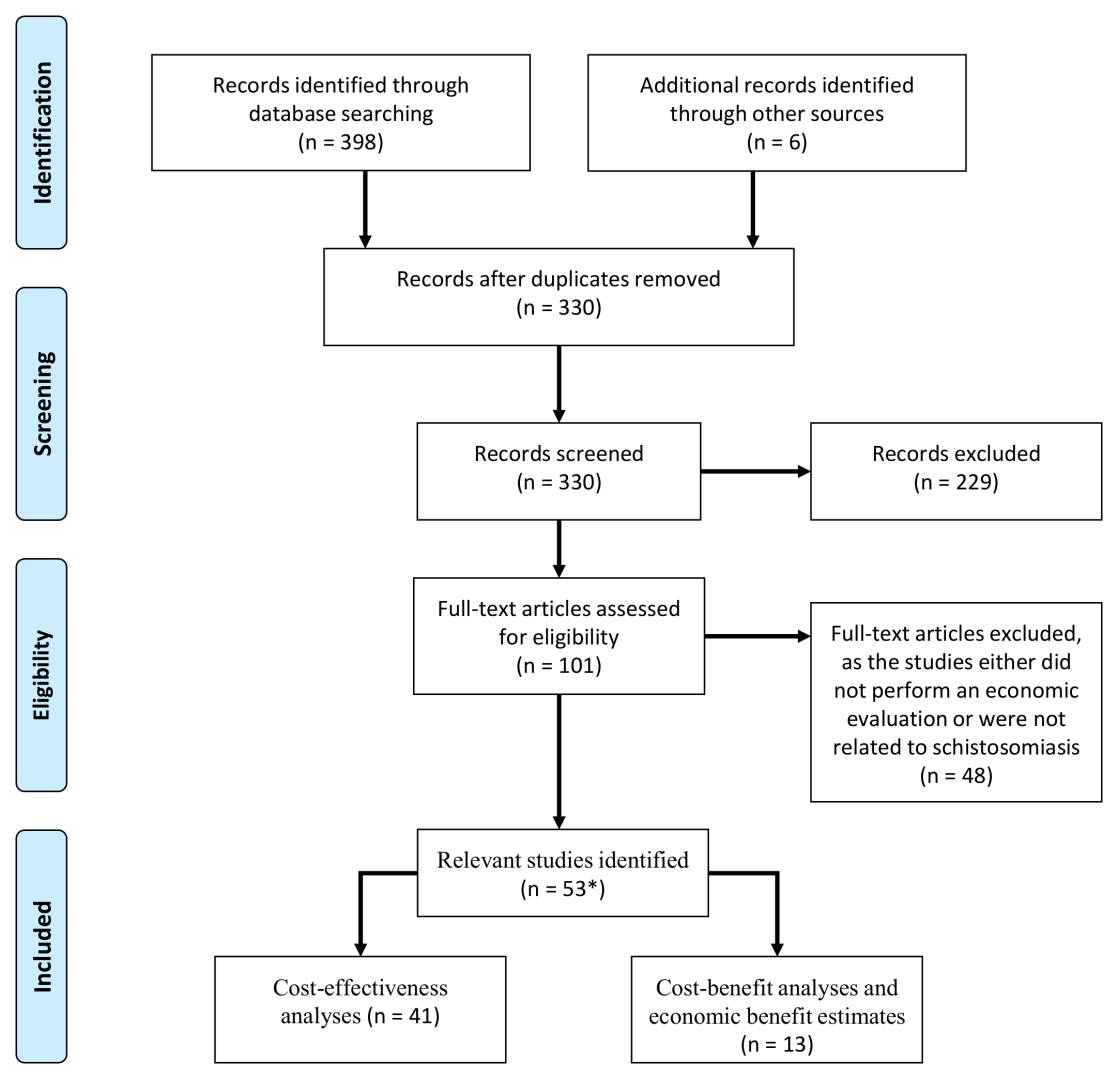
Flow diagram outlining the inclusion and exclusion of the identified studies. *Some studies reported both cost-benefit and cost-effectiveness estimates. A PRISMA checklist is supplied as extended data ^15^.

The titles and abstracts of the identified papers were examined initially for relevance by two independent reviewers [HCT and JT]. The full texts for potentially relevant articles were then reviewed to determine eligibility for inclusion. The bibliographies of papers suitable for inclusion were then scanned for studies not originally retrieved from the databases. Discrepancies were solved by consensus among the reviewers. The full selection process is outlined in Figure 1.

The identified health economic studies were grouped into two broad types:

- Cost-effectiveness analyses and cost-utility analyses: Cost-effectiveness analysis is a form of economic analysis that compares the relative costs and effectiveness of different courses of action. The effectiveness of the interventions under investigation is measured in terms of natural units (such as life years gained, cases averted, or heavy cases averted). Cost-utility analysis is a specific subtype of cost-effectiveness analysis, where the effectiveness of the intervention is measured using a "utility-based" unit (such as disability-adjusted life years (DALYs) averted and quality-adjusted life years (QALYs) gained) ( Box 1).
- Cost-benefit analyses and estimates of economic benefits: Cost-benefit analysis is a form of economic analysis that compares the relative costs and benefits of different courses of action ( Box 1). Unlike, cost-effectiveness analyses, the benefits of an intervention are expressed in monetary terms, i.e. compares the cost of an intervention to its monetary benefits. We also included studies that only estimated the economic benefits of schistosomiasis interventions.

Due to the number of studies identified and the range of research questions investigated ( Figure 1), it was not possible to provide a detailed summary of the results of every study. Instead in line with the aim of the paper, we provide an overview of the studies that have been done and discuss key overarching findings and research gaps that need to be addressed. Due to their significance to policy makers, we outline further details of the studies reporting the cost per DALY averted related to preventive chemotherapy.

The studies estimates were kept in their original cost year and not adjusted to a single reference year.

## Results

We identified 53 relevant health economic analyses of schistosomiasis interventions. An overview of the studies is presented in Table 1. Interestingly, there were notably more economic evaluations of schistosomiasis than those identified in previous reviews for other helminth infections of human health importance ^16– 18^.

**Figure 2.**
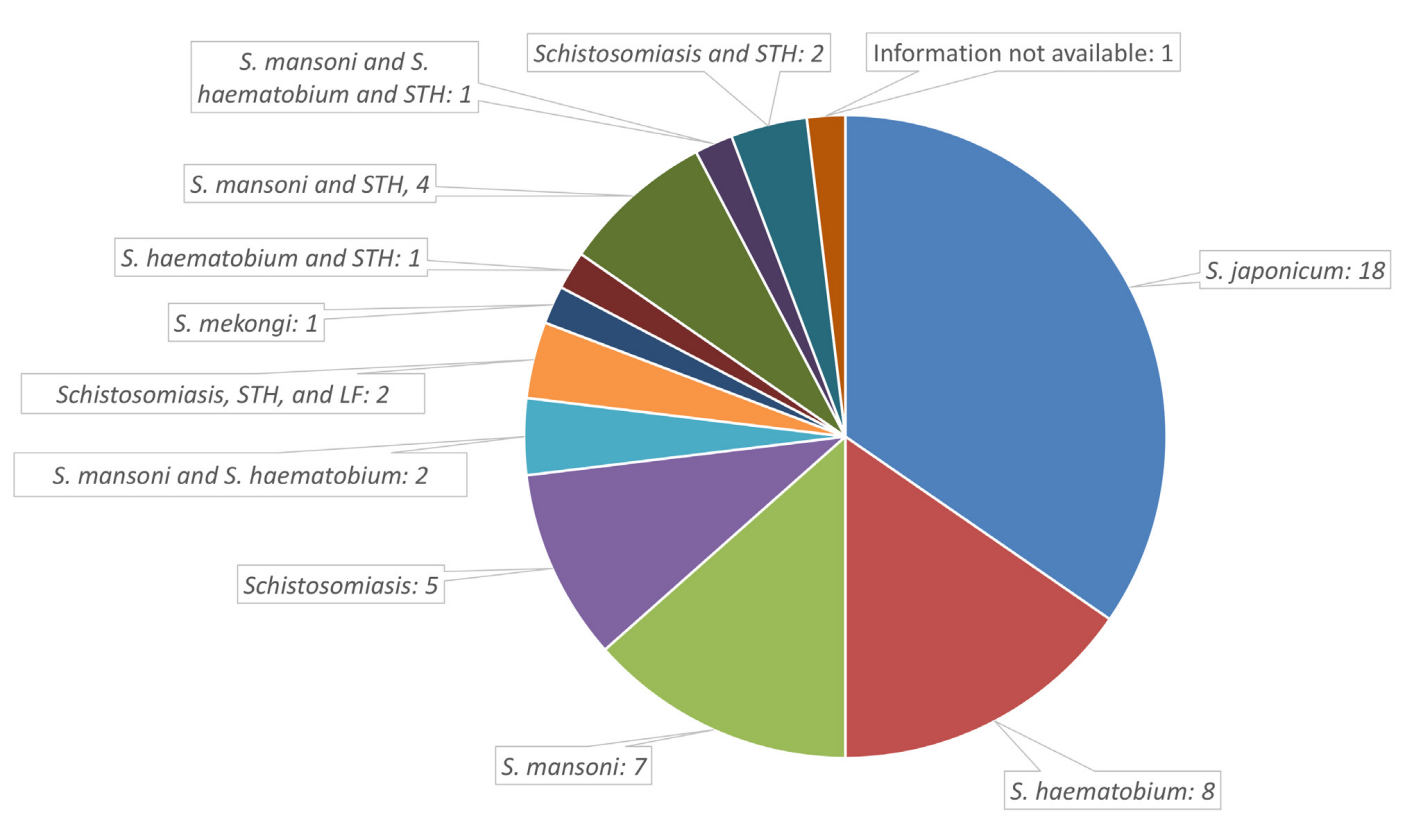
Overview of the number of studies done and the investigated species. LF: Lymphatic filariasis, STH: Soil-transmitted helminths.

The number of economic evaluations performed on schistosomiasis interventions has gradually increased over time, although a notable number were published pre-2000 ( Table 1). Most studies were related to *S. japonicum,* followed by *S. haematobium,* and *S. mansoni* ( Figure 2). Only one study related to *S. mekongi* ( Table 1). Several studies also included other NTDs, particularly soil-transmitted helminths (STH). In Africa, most studies evaluated mass preventive chemotherapy, whereas in China they mostly evaluated programmes using a combination of interventions together ( Table 1). Few studies were performed for other settings ( Table 1).

**Table 1.** Summary of the identified studies.

Study	Publication year	Species	Intervention(s)	Research focus
Cost-effectiveness analyses				
19	1987	Schistosomiasis	PC	The optimal choice of PC strategy (selective vs mass).
20	2006	Schistosomiasis	PC	The cost-effectiveness of treating school-age children for schistosomiasis.
21	2011	Schistosomiasis	PC	The cost-effectiveness of PC for schistosomiasis control.
22	1977	*S. haematobium*	Chemotherapy and snail control (separately and in combination)	The cost-effectiveness of alternative disease control measures within the Khuzestan Province, Iran.
23	1986	*S. haematobium*	Selective chemotherapy	The cost-effectiveness of selective chemotherapy; 1) metrifonate - 3 dose regimen, fortnightly intervals vs 2) praziquantel - one dose regimen
24	1994	*S. haematobium*	Alternative treatment strategies	The cost-effectiveness of alternative treatment strategies; 1) Mass treatment by a mobile team, 2) Reagent strip testing by schoolteachers with referral to the dispensary for treatment, 3) Passive testing and treatment at the dispensary
25	2011	*S. haematobium*	PC	The cost-effectiveness of PC for schistosomiasis control in Niger.
26	2013	*S. haematobium*	PC	The potential cost-effectiveness of schistosomiasis treatment for reducing HIV transmission in Africa.
27	2013	*S. haematobium*	Provision of clean water, sanitation, and health education with administration of praziquantel to school-aged children	The cost-effectiveness of a community-based intervention for reducing the transmission of *S. haematobium* and HIV in Africa.
28	2018	*S. haematobium*	PC and/or snail control	The cost-effectiveness of PC, focal chemical-based snail control, and combined strategies against schistosomiasis.
29	2000	*S. japonicum*	Selective chemotherapy	The cost-effectiveness of mass indirect hemagglutination screening vs. a questionnaire followed by indirect hemagglutination testing.
30	2001	*S. japonicum*	Selective chemotherapy vs PC	The cost-effectiveness of selective chemotherapy (using water contract surveys) vs PC in Hunan province, China.
31	2002	*S. japonicum*	Chemotherapy	The cost-effectiveness of three chemotherapy schemes against *S. japonicum* in Dongting Lake region, China; 1) Treatment to those with contact with infected water and/or symptoms of infection; 2) mass chemotherapy-treatment; and 3) treatment prescribed to positive cases after Kato-Katz examination
32	2002	*S. japonicum*	Information not available	Information not available.
33	2003	*S. japonicum*	Information not available	The cost-effectiveness of a rapid control strategy in new hilly endemic areas of schistosomiasis in Taoyuan County, China.
34	2003	*S. japonicum*	A combination of interventions	The cost-effectiveness and benefit analysis of World Bank Loan on schistosomiasis control in Qiangjiang City, China.
35	2005	*S. japonicum*	A combination of interventions	The cost-effectiveness of the national schistosomiasis control programme in China from 1992 to 2000.
36	2009	*S. japonicum*	A combination of interventions	The cost-effectiveness of a more intensive strategy vs routine interventions in the Poyang Lake region, China.
37	2011	*S. japonicum*	Snail control - environmental modification	The cost-effectiveness of a snail control project using environmental modification in hilly regions, China.
38	2013	*S. japonicum*	A combination of interventions	The cost-effectiveness of a comprehensive *Schistosomiasis* *japonica* control programme in the Poyang Lake region, China.
39	2013	*S. japonicum*	Information not available	The cost-effectiveness of schistosomiasis comprehensive control in Lushan County, China from 2007 to 2012.
40	2014	*S. japonicum*	A combination of interventions	The cost-effectiveness of comprehensive control measures carried out in schistosomiasis endemic inner embankment of marshland and lake regions from 2006 to 2010 in Jiangling County, China.
41	2017	*S. japonicum*	A combination of interventions	The cost-effectiveness of a comprehensive schistosomiasis control strategy with a focus on cattle and sheep removal in Junshan District, China.
42	2018	*S. japonicum*	Snail control – molluscicides	The cost-effectiveness of three molluscicides in Yangtze River, China.
43	1977	*S. mansoni*	Snail control, chemotherapy, and provision of water supplies	The cost-effectiveness of three different schistosomiasis interventions.
44	1984	*S. mansoni*	Chemotherapy with oxamniquine with and without snail control	The cost-effectiveness of different ways of controlling intestinal schistosomiasis in the mining region of Maniema in Eastern Zaire.
45	1995	*S. mansoni*	Vaccination (hypothetical)	The desirable characteristics of a schistosomiasis vaccine.
46	1997	*S. mansoni*	Vaccination (hypothetical)	The target product profile of a schistosomiasis vaccine.
47	1998	*S. mansoni*	PC	An investigation into the interaction between drug efficacy and drug price of praziquantel in determining the cost- effectiveness of school-based schistosomiasis treatment.
48	2000	*S. mansoni*	Treatment at a primary health care centre	The cost-effectiveness of different treatment strategies at primary health care centres in Burundi; 1) screening all symptomatic patients (with Kato-Katz) and treating positive cases; 2) treating all symptomatic patients or 3) treating only those presenting with symptoms of severe diarrhoea.
49	2010	*S. mekongi*	PC	The cost-effectiveness of the schistosomiasis control programme in Cambodia.
50	1993	Schistosomiasis and STH	PC	The cost-effectiveness of school-based treatment (via a mobile team) for schistosomiasis and STH.
51	2000	*S. mansoni* and *S. haematobium*	PC	The impact of school attendance on the unit cost and effectiveness of school-based schistosomiasis chemotherapy programmes.
52	2001	*S. haematobium* and STH	PC	The impact and cost-effectiveness of school-based anthelmintic treatments in reducing anaemia in children in the United Republic of Tanzania.
53	2004	*S. mansoni* and STH	PC	The cost-effectiveness of a Kenyan school-based PC project.
54	2008	Schistosomiasis and STH	PC	The cost-effectiveness of nationwide school-based helminth control in Uganda: intra-country variation and effects of scaling-up.
55	2011	*S. mansoni* and *S. haematobium*	PC	A life path analysis to estimate the incremental cost- effectiveness of using double treatment (given 2 to 8 weeks after the first dose) instead of single annual treatment in school-based and community-wide PC programmes.
56	2015	*S. mansoni* and *S. haematobium* and STH	PC	A comparison of the cost-effectiveness of school-based vs community-wide PC for schistosomiasis and STH.
57	2016	*S. mansoni* and STH	PC	An assessment of the global guidelines for PC strategies against schistosomiasis and STH.
58	2018	Schistosomiasis, STH, and LF	PC	The cost-effectiveness of PC in Madagascar.
59	1996	Information not available	Vaccination (hypothetical)	The potential cost-effectiveness of a vaccine against schistosomiasis.
**Cost-benefit analyses and estimates of economic benefits**				
60	2000	Schistosomiasis	Various preventive schistosomiasis interventions	The cost-benefit of different preventive schistosomiasis interventions in Kenya.
61	2017	Schistosomiasis	PC	The socioeconomic benefit of achieving the WHO 2020 targets for schistosomiasis.
62	1974	*S. haematobium*	Not applicable	The potential economic benefits of eliminating mortality attributed to schistosomiasis in Zanzibar.
63	1987	*S. japonicum*	Mass chemotherapy and snail control (molluscicides)	The investigation of a theoretical model to determine the most cost-efficient combination of preventive and curative measures in China.
35	2005	*S. japonicum*	A combination of interventions	An economic evaluation of the national schistosomiasis control programme in China from 1992 to 2000.
36	2009	*S. japonicum*	A combination of interventions	The cost-benefit of a more intensive strategy vs routine interventions in the Poyang Lake region, China.
64	2011	*S. japonicum*	Snail control – forest environment	The eco-economical benefit of snail control and schistosomiasis prevention in mountainous regions in Yunnan Province, China.
65	2012	*S. japonicum*	Snail control – environmental modification vs molluscicides	The cost-benefit of snail control by environmental modification (such as building low dykes, ploughing and planting) vs. molluscicide use in Jiaobei Beach of Zhenjiang City, China.
66	1972	*S. mansoni*	Snail control and mass diagnosis/treatment campaign	A cost-benefit analysis of an *S. mansoni* control programme on an irrigated sugar estate in northern Tanzania.
53	2004	*S. mansoni* and STH	PC	The economic benefits of a school-based PC project in Kenya.
67	2016	*S. mansoni* and STH	PC	The economic benefits of a school-based PC project in Kenya.
58	2018	Schistosomiasis, STH, and LF	PC	The financial, and education gains of investing in preventive chemotherapy in Madagascar.

LF: Lymphatic filariasis, STH: Soil-transmitted helminths PC: Preventive chemotherapy.

The methods used by the studies to parameterise the costs of preventive chemotherapy were variable: some used primary cost data whereas others performed rough calculations or used assumed crude benchmark values ( Table 2). There was also variation regarding the use of financial or economic costs within the studies ( Box 1). The economic cost of an intervention is typically higher than the financial cost ( Table 2). If praziquantel was assumed to be purchased it would be counted as a financial cost. Depending on the perspective of the analysis, the value of any donated praziquantel would be included as an economic cost. It should be noted that the shift towards drug distribution being integrated within school systems reduced the delivery costs associated with preventive chemotherapy. Consequently, the results of many of the earlier studies cannot be directly generalised to current control programmes ( Table 2).

**Table 2.** Cost-utility analyses of preventive chemotherapy for schistosomiasis.

Study	Setting	Time horizon for the effectiveness	Intervention	Assumed average costs of preventive chemotherapy	Cost-effectiveness ratio or incremental cost-effectiveness ratio	Cost year
Schistosomiasis alone						
Hotez *et al.* (DCP2) ^20^	Hypothetical setting (Schistosomiasis)	Not clearly stated	Annual mass school-based treatment	Not stated	US$336–692 per DALY averted (note that this is incorrectly quoted as US$3.36–6.92 within the report)	Unclear
GiveWell ^21^	Hypothetical setting (Schistosomiasis)	1 year	Annual mass school-based treatment	US$0.27-0.47 per treatment (including drug costs)	US$30–$80 per DALY averted	Unclear
Lo *et al.* ^57^	Hypothetical setting ( *S. mansoni)*	5 years	Annual mass school-based treatment	US$0.71 per treatment (including drug costs)	5% prevalence in SAC: US$1,050 per DALY averted, 15% prevalence in SAC: US$449 per DALY averted, 30% prevalence in SAC: US$160 per DALY averted	2015 prices
			Annual mass community- wide treatment	US$1.71 per treatment (including drug costs)	15% prevalence in SAC: US$1,031 per incremental DALY averted, 30% prevalence in SAC: US$443 per incremental DALY averted	
King *et al.* ^55^	Kenya *(S. mansoni and* *S. haematobium)*	Liftime	Annual mass school-based treatment	Delivery cost: US$0.811 per treatment (including drug costs)	*S. mansoni*: US$17.76 per QALY gained *S. haematobium*: US$17.18 per QALYgained	Unclear
			Annual mass school-based treatment (double treatment)	Delivery cost: US$0.811 per treatment (including drug costs)	*S. mansoni*: US$152.95 per incremental QALY gained *S. haematobium*: US$210.83 per incremental QALY gained	
			Annual mass community- wide treatment	Delivery cost: US$0.811 per treatment (drug costs were also included)	*S. mansoni:* US$47.90 per QALY gained *S. haematobium*: US$45.58 per QALY gained	
			Annual mass community-wide treatment (double treatment)	Delivery cost: US$0.811 per treatment (drug costs were also included)	*S. mansoni:* US$291.07 per incremental QALY gained *S. haematobium*: US$432.76 per incremental QALY gained	
Ndeffo Mbah *et al.* ^27^	Zimbabwe *(S. haematobium)*	Lifetime	Provision of clean water, sanitation, and health education with annual administration of praziquantel to school-aged children	US$0.41 per treatment (including drug costs) Costs of clean water, sanitation, and health education were varied.	-	Unclear
Schistosomiasis and soil-transmitted helminths (STH):						
Lo *et al.* ^56^	Four communities in Côte d'Ivoire (Schistosomiasis)	15 years	Annual mass school-based treatment	US$0.71 per treatment (including drug costs)	US$118 (US$87–141) per DALY averted (92% of the disability resulted from *Schistosoma* infections)	2014 prices
			Annual mass community-wide treatment for schistosomiasis (biannual for STH)	US$1.71 per treatment (including drug costs)	US$167 (US$101 to 463) per incremental DALY averted	
Miguel and Kremer ^53^	Kenya ( *S. mansoni* and STH)	1 year	Annual mass school-based treatment	US$0.49 per pupil per year (including drug costs)	US$5 per DALY averted (99% of the benefit was due to averted schistosomiasis)	Unclear
Warren *et al.* ^50^	Hypothetical setting (Schistosomiasis and STH)	10 years	Annual mass school treatment with a mobile team	US$0.8–1.80 per child per year (including drug costs)	US$6–33 per DALY averted	Unclear

DALY: Disability-adjusted life year, QALY: Quality-adjusted life year, SAC: School-aged children STH: Soil-transmitted helminths.

### The cost-effectiveness analyses

We identified 41 cost-effectiveness analyses of schistosomiasis interventions ( Table 1). The majority investigated preventive chemotherapy, with few looking at the cost-effectiveness of water, sanitation and hygiene (WASH) and behavioural change ( Table 1). A range of effectiveness metrics were used by the different studies, including specific types of morbidity averted (such as anaemia), cases prevented, heavy infections averted, decreases in the infection rate (in humans, animal hosts and snails) and DALYs averted.

One of the key areas of analysis was whether selective treatment should be used i.e. where only those that are tested positive for infection (or suspected to be infected) are treated. This strategy uses less praziquantel relative to mass preventive chemotherapy. Some earlier studies found that selective treatment could be more cost-effective than mass treatment ^24, 48^. However, as the price/value of praziquantel and delivery declined over time, mass school-based or community-wide treatment became more cost-effective than using screening/selective based approaches, particularly in high prevalence settings ^19, 31, 48^. By adopting cheaper methods of performing selective treatment, such as water contact surveys rather than testing for infection with diagnostics, selective treatment may become more cost-effective in certain settings ^30, 68^.

The estimated cost per DALY averted for annual mass school-based preventive chemotherapy for schistosomiasis (or both STH and schistosomiasis) ranged widely between US$5-692 in moderate and high prevalence settings ( Table 2– cost year variable) ( Box 1). In many settings, annual school-based preventive chemotherapy would be classed as cost-effective (depending on the cost-effectiveness threshold ( Box 2)). Lo *et al.* ^57^ demonstrated that the cost-effectiveness of annual school-based preventive chemotherapy was highly influenced by the setting’s prevalence of schistosome infections (5% prevalence in SAC: US$1,050, 15% prevalence in SAC: US$449, and 30% prevalence in SAC: US$160 (2015 prices)). This shows that in moderate to high prevalence settings ( Box 1), annual preventive chemotherapy is generally cost-effective supporting the WHO recommendation of less frequent treatment in low prevalence settings. The range in these estimates was also influenced by the method used for calculating the number of DALYs averted, particularly the choice of disability weight. Community-wide preventive chemotherapy tended not to be more cost-effective than school-based treatment for schistosomiasis control ^55– 57^, i.e. it did not have a lower cost per DALYs averted. However, it could be classed as cost-effective depending on the cost-effectiveness threshold ( Box 2). De Neve *et al.* ^58^ highlighted that the overall cost-effectiveness of preventive chemotherapy depends on which other infections are co-endemic.

Box 2. **Cost-effectiveness thresholds**

To determine whether an intervention is cost-effective using a cost-utility analysis, the cost per DALY averted is compared to a cost-effectiveness threshold. An often misunderstood aspect of cost-utility analysis is that when comparing mutually exclusive interventions (such as school-based vs community-wide preventive chemotherapy), the goal is to find the most effective intervention which has an incremental cost-effectiveness ratio below an established cost-effectiveness threshold. It is not about finding the intervention/strategy with the lowest cost-effectiveness ratio (i.e. the strategy with the lowest cost per DALY averted). The findings of cost-utility analysis are often miscommunicated to policymakers and many refer to an intervention being the “most cost-effective”, when instead they mean it is the optimal intervention for the given cost-effectiveness threshold.

The most appropriate cost-effectiveness thresholds are under debate within the global health field ^69– 71^. Some studies used the cost-effectiveness threshold set by the WHO-CHOICE ^72^, namely a cost per DALY averted < 3 times the country’s GDP per capita. However, this is now considered to be too high and has been widely criticised ^69– 71, 73, 74^. Interestingly, recent analyses have indicated that a significantly lower cost-effectiveness threshold closer to < ½ the country’s per capita GDP would be more appropriate for low-income countries ^73, 75^. The Disease Control Priorities project (Third Edition) also used a more conservative threshold of US$200 per DALY averted to identify priority interventions for consideration in low-income countries ^76^. These different thresholds are shown in the Table below. It is vital that studies are interpreted in light of such changes and reduced thresholds – as conclusions regarding what interventions or strategies are cost-effective may no longer hold. This will be an important consideration when using cost-effectiveness analysis to inform new schistosomiasis treatment guidelines, particularly guidance on the use of community-wide treatment.

**Table:** Cost-effectiveness thresholds recommended for low-income countries.

Threshold source	Average cost per DALY averted threshold (2017 prices)
Previous WHO threshold ^72^	US$2,355 (highly cost effective: US$785)
Proposed update to WHO threshold ^73, 75^	US$392.5
World Bank ^77^	US$235.50 *

* The value has been adjusted to 2017 prices using GDP implicit price deflators relating to US$ ^78^. DALY: disability-adjusted life year, WHO: World Health Organization, GDP: Gross Domestic Product.

Most of the studies related to *S. japonicum* in China evaluated programmes using a combination of interventions together (such as chemotherapy, snail control and health education). These studies often looked at one specific setting whilst evaluating the currently used interventions, though some evaluated more comprehensive interventions ^36^ or alternative strategies ^30, 31^. Many of these studies investigated the cost per reduction in the infection rate, often considering humans, animal hosts and snails ( Table 1). For example, Zhou *et al.* ^35^, performed a retrospective economic evaluation of the national schistosomiasis control programme in China from 1992 to 2000. Based on data derived from the six study counties, they estimated that the average cost for case detection was 12.48 Chinese yuan per person (cost year varied). The average costs to reduce the human infection rate of *S. japonicum* per 100 persons by 1% was 7732.42 Chinese yuan (cost year varied) and the bovine *S. japonicum* infection rate per 100 cattle by 1% was 162891.10 Chinese yuan (cost year varied). Reducing the snail-infested areas by 1000m ^2^ by mollusciciding costed 3573.18 Chinese yuan (cost year varied). Due to the contrasting effectiveness metrics, it is difficult to make comparisons to the studies on other settings or diseases.

Most studies evaluating snail control were in China and looked at several types of control method, including environmental modification and molluscicide use. These were typically part of a programme and not a standalone intervention. It is critical not to overgeneralise studies regarding the impact and cost-effectiveness of snail control. For example, the benefit of snail control will likely be higher for zoonotic species such as *S. japonicum.* Only Lo *et al.* ^28^ investigated the cost-effectiveness of snail control for controlling *S. haematobium* using DALYs as the effectiveness metric. Their results supported the use of snail control within schistosomiasis control strategies, particularly in high prevalence settings, transmission hotspots, and settings with high noncompliance to preventive chemotherapy ^28^.

King *et al.* ^55^ evaluated the likely cost-effectiveness of giving repeated (or double) treatment, whereby two rounds of treatment are provided 2 to 8 weeks apart to enhance impact. Using Markov modelling of potential lifetime gains, they found that although schedules for repeated treatment with praziquantel require greater inputs in terms of direct costs and community participation, there are incremental benefits to this approach at an estimated incremental cost of US$153 ( *S. mansoni*) – US$211 ( *S. haematobium*) per QALY gained when repeated treatment is employed within school-based programmes.

Ndeffo Mbah *et al.* ^26^ performed a cost-effectiveness analysis of annual administration of praziquantel to SAC as a potential measure to reduce the burden of HIV in sub-Saharan Africa. Epidemiological data have shown that genital infection with *S. haematobium* – known as female genital schistosomiasis (FGS) - increases the risk of HIV infection in young women ^79, 80^. They found that praziquantel administration could potentially be a cost-effective and even cost saving way of reducing HIV infections in sub-Saharan Africa, particularly where *S. haematobium* and HIV are highly prevalent. Programme costs per case of HIV averted were similar to, and under some conditions better than, other interventions that are currently implemented in Africa to reduce HIV transmission. These cost-savings occurred due to the low cost and high efficacy of praziquantel, along with the increased HIV risk faced by women infected with FGS. In addition to FGS, schistosome infections have also been associated with increased transmission of HIV in males ^81^.

A further study by Ndeffo Mbah *et al.* ^27^ evaluated the cost-effectiveness of a community-based intervention for averting *S. haematobium* infections and resultant HIV. The intervention integrated the provision of clean water, sanitation, and health education (WSHE) for the entire community with annual praziquantel treatment of SAC. The cost-effectiveness of the community-based intervention was found to vary with the cost of WSHE, the efficacy of WSHE for reducing *S. haematobium* transmission and the duration of the intervention. The intervention remained cost-effective for a range of WSHE efficacies and became more cost-effective over time. Overall, the results indicated that this integrated community-based approach towards schistosomiasis control could effectively reduce the health and economic burden associated with *S. haematobium* and HIV infections in sub-Saharan Africa.

### The cost-benefit analyses and estimates of economic benefits

Schistosomiasis can be debilitating and can negatively impact productivity ^2, 67, 82– 91^. A recent systematic review and meta-analysis found that *Schistosoma* infection/non-treatment was significantly associated with educational, learning and memory deficits in SAC ^92^.

A number of studies have estimated the economic benefits or performed cost-benefit analyses of schistosomiasis interventions ( Table 1). For example,

- Redekop *et al.* ^61^ estimated US$17.4 billion in economic benefit would be generated (between 2011–2013) if the WHO 2020 roadmap goals for schistosomiasis were achieved (2010 prices). The majority of this benefit was due to prevented anaemia.
- De Neve *et al.* ^58^ estimated the health, financial, and education gains of investing in preventive chemotherapy for schistosomiasis, STH, and lymphatic filariasis in Madagascar. They found that preventive chemotherapy could avert a notable amount of school absenteeism and reduce patients’ out-of-pocket expenditure.
- Zhou *et al.* ^35^ investigated the cost-benefit of the national schistosomiasis control programme in China (1992–2000). The net benefit-cost ratio was 6.20.
- Miguel and Kremer ^53^ found that deworming for STH and schistosomiasis was likely to increase the net present value of wages by over US$30 per treated child based on the estimated rate of return to education in Kenya. Baird *et al.* ^67^ also subsequently estimated that mass deworming may generate more in future government revenue than it costs in subsidies.

These studies indicate that schistosomiasis interventions may generate notable economic benefits. These types of analyses were less common compared to cost-effectiveness analyses. This is likely due to the methodological challenges of placing a monetary value on the benefits of schistosomiasis interventions. There was notable variation regarding the methodology of these identified studies and what was used to justify the assumptions made. It should be highlighted that the results of such studies can be highly sensitive to the methodology used. Further standardisation is urgently needed in this area (not just for studies on schistosomiasis).

## Discussion

We identified a wide range of economic evaluations of schistosomiasis interventions. Due to the variation in methodology and epidemiological settings, it was not possible to make definitive policy recommendations based on the identified studies. However, the results of the review indicate that annual schistosomiasis treatment interventions are generally estimated to be cost-effective in moderate and high prevalence settings ( Box 1) and can generate notable economic benefits. In low prevalence settings aiming for morbidity control or elimination as a public health problem ( Box 1), annual mass preventive chemotherapy may not be cost-effective, supporting the current WHO recommendation of less frequent treatment in such settings ^10^. There are also a growing number of studies evaluating alternative strategies to those currently recommended, such as snail control and WASH.

Most of the studies related to *S. haematobium* and *S. mansoni* evaluated mass preventive chemotherapy. Contrastingly, most of the studies relating to *S. japonicum* evaluated comprehensive control programmes with multiple components (such as snail control, preventive chemotherapy, health education). These studies were difficult to compare with others as they tended to use metrics based on reductions in infection rates (humans, animal hosts and snails) rather than DALYs or cases/morbidity averted.

There was notable variation in methodology across the different studies (which likely lead to the wide range in the estimated costs per DALY averted ( Table 2)). Key sources of variation included 1) the assumed costs, 2) the epidemiolocal setting, and 3) the methods used to quantify the effectiveness of an intervention.

### Variation in the assumed cost of interventions

The average delivery cost of annual preventive chemotherapy using praziquantel was typically assumed (or in some cases estimated) to be between US$0.20-0.50 per treatment ^93^. This is consistent with a recent systematic review by Salari *et al.* ^94^, which found that the average delivery cost of preventive chemotherapy for schistosomiasis (with or without an educational component) was US$0.30 per treatment. However, the methods used to parameterise the costs of different interventions within these economic evaluations varied widely making it difficult to understand the relative costs of different interventions and how costs may vary across countries/regions. This variation also made it difficult to directly compare the different studies. Salari *et al.* ^94^ also found that the degree of transparency for most of the costing studies of schistosomiasis interventions they identified was limited. An important consideration is whether financial or economic costs are being used ( Box 1). Economic costs are considered the gold standard within economic evaluations as they better reflect the sustainability and replicability of interventions. Many studies did not formally state if they were using financial or economic costs. There was also variation regarding whether the cost of donated praziquantel had been included within the analysis as an economic cost ( Table 2).

When interpreting and comparing the studies, it is vital to consider that integrating drug distribution through the school system rather than using mobile teams notably reduced the delivery costs associated with preventive chemotherapy. However, many of the earlier health economic studies identified (pre-2000) related to the use of mobile teams to deliver treatments, which is more costly than the currently used school-based platform. Additionally, in the past, the cost of praziquantel was substantially higher ^47, 95^ with many of the studies having praziquantel costing US$0.60 per treatment. However, several forms of generic praziquantel tablets are now available, currently costing around US$0.08 per tablet ^96, 97^ and some countries (such as China ^95, 98^) produce their own. Merck KGaA have also committed to donate 250 million tablets of praziquantel annually (primarily for SAC in Africa) ^10^. Consequently, the findings of many of the earlier studies cannot be directly generalised to current control programmes.

### Variation in the epidemiological setting

Pre-control endemicity is a notable driver in the estimated cost-effectiveness of schistosomiasis interventions. As pre-control endemicity increases, the intervention generally becomes more cost-effective. This needs to be considered when comparing studies and using them to inform policy.

**Figure 3.**
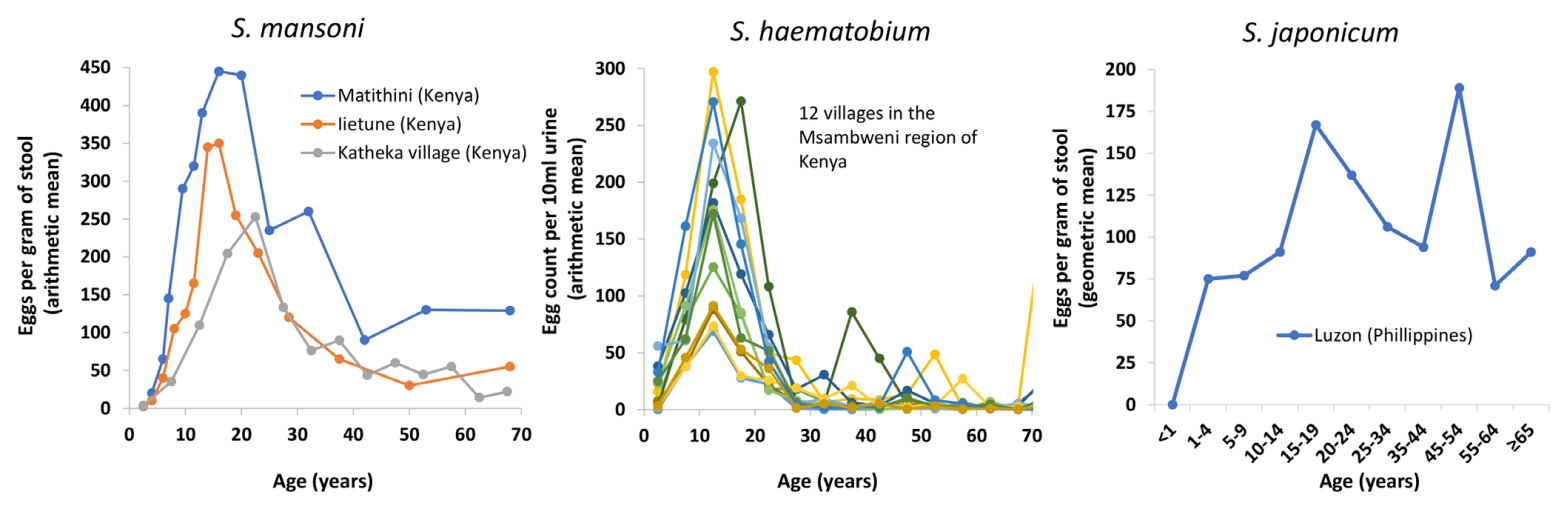
The observed cross-sectional host-age and mean infection intensity profiles for schistosome infections. The data are from the following sources: Iietune village, Kenya ^110^, Matithini village, Kenya ^110^, Katheka village, Kenya ^111^, 12 villages in the Msambweni region of Coastal Kenya ^112^, and Luzon, Philippines ^113^. Note that these show the mean infection intensity for the entire sampled population (i.e. including both infected and non-infected individuals).

In contrast, the importance of the age profile of infection is more subtle and often ignored. Although the assumed age profile of infection would have a relativity small impact on the estimated cost-effectiveness of the recommended school-based treatment, it can have notable implications regarding the relative benefit of alternative interventions, particularly regarding the benefit of targeting additional age groups with preventive chemotherapy. Consequently, it needs to be considered in studies investigating the cost-effectiveness of alternative schistosomiasis interventions to school-based treatment ( Table 3) and has substantial consequences regarding the generalisability of economic evaluations and their conclusions. Figure 3 illustrates some of the available age profile data. It should be noted that the shape of the typical age profile (and variation around them) differs for each species. The importance of these age profiles of infection and their variation is often not accounted for in modelling studies and policy recommendations ^99^. There is a real danger that overgeneralising in this area could lead to highly inefficient recommendations. Caution is needed when parameterising this aspect of models when performing economic evaluations. For example, if the data relating to adults are not representative, such as being from high-risk adults only, the assumed average burden of adult infection and cost-effectiveness of switching to community-wide treatment will be overestimated ^100^.

**Table 3.** Age profiles of infection assumed in previous modelling studies on *Schistosoma mansoni*.

Study	Measure	Mean burden in Pre-SAC	Mean burden in SAC	Mean burden in adults	Sources
Chan *et al.* and Guyatt *et al.* ^47, 101– 103^	Peak age of water contact (mean EPG at peak)		15 years old (EPG: 300 and 500)		47, 101
Lo *et al.* ^56^	EPG – four different communities were modelled	37.1 6.1 5.1 0	94.2 229.5 30.1 1.1	138.9 446.9 89.6 4.8	104 105, 106 107 108
Lo *et al.* ^57^	Relative prevalence by age group	0.625	1	0.8333	109
Turner *et al.* ^99^	EPG for three scenarios with low, moderate and high relative adult burdens *	Higher transmission setting: 3 12.3 3.1 Lower transmission setting: 1.2 4.8 1.2	Higher transmission setting: 308.7 267.3 215.4 Lower transmission setting: 157.7 133.3 101.5	Higher transmission setting: 149.2 184.4 232.1 Lower transmission setting: 71.6 88.8 114.4	110, 111

Pre-SAC: pre-school-aged children (2–4 years old), SAC: school-aged children (5–14 years old), adults: 15+ years old, EPG: eggs per gram. Note that these show the mean infection intensity for the entire age group (i.e. including non-infected individuals). * The values from Turner *et al.* ^99^ were converted from worm burden to EPG.

A further factor is whether other species are included within the economic evaluation ( Table 1 and Table 2). Based on the available studies, we cannot make inferences on the relative cost-effectiveness for different species. Generally, accounting for other species would increase the cost-effectiveness or cost-benefit of preventive chemotherapy. In addition, Lo *et al.* highlighted that the optimal strategy for schistosomiasis can depend on if it is co-endemic with STH ^57^.

### Variation in the methods used to quantify the effectiveness of interventions

A key area of uncertainty and variation is how the health gains/effectiveness of interventions were quantified. This is important as the chosen method impacts the outcome of an economic evaluation, potentially leading to different policy recommendations. For example, when basing effectiveness on the number of worm years or heavy cases years averted (both metrics related to infection intensity), models find that the total effectiveness tends to increase with the transmission setting ^99^. In contrast, when the effectiveness is based on reductions in the prevalence of infection, the estimated effectiveness can be greater in lower transmission settings ^99^. This would imply that when modelling reductions in morbidity are based on reductions in prevalence, the results could find that it is more cost-effective to treat in lower transmission settings – which is likely to be misleading in terms of morbidity control. The effectiveness metric also has a particularly notable influence on the relative benefit of annual community-wide to school-based treatment: metrics based on reduction in infection intensity tend to estimate a lower benefit relative to those based on infection prevalence ^99^.

Typically, the ideal metric for evaluating different control strategies in low- and middle-income countries is the number of DALYs averted ( Box 1). As this metric is used for a wide range of diseases, the cost-effectiveness estimates can be directly compared to other healthcare interventions. This makes it possible to have standardised thresholds to class whether an intervention is cost-effective ( Box 2) which is rarely possible for disease-specific metrics. However, the DALY burden of schistosomiasis is often calculated by simply applying a disability weight (representing the disability of an `average' prevalent case of schistosomiasis) to the prevalence of infection. Although this approach may be a suitable method for approximating the disease burden of schistosomiasis at a given point in time, we would argue that it is misleading to apply this framework to evaluate the effectiveness of schistosomiasis interventions ^99^. This is because the relationship between schistosome infection and morbidity is complex and often not merely due to the presence or absence of infection ^99, 114^. Only quantifying health gains based on reductions in prevalence would implicitly assume that curing a very light infection has a health benefit whereas reducing a heavy infection to a light infection has none.

The DALY disability weights have also been controversial for schistosomiasis (defined in terms of 0 being perfect health and 1 being death ( Box 1)) ^2, 115, 116^. The Global Burden of Disease (GBD) 1990 study gave schistosomiasis a disability weight of 0.005 ^117^, implying a very minimal level of disability for “average” schistosomiasis. In contrast, a higher weight of 0.02 was proposed by King *et al.* ^2, 118^. The GBD now has an average weight of 0.006 for mild infection, with higher disability weights for specific forms of morbidity (such as anaemia, bladder pathology, and haematemesis) ^119^. The notable variation in the methods used to estimate DALYs related to schistosomiasis makes it difficult to directly compare the results of different studies reporting a cost per DALY averted for schistosomiasis interventions ( Table 2). It is vital that this is recognised when interpreting these studies.

King *et al.* ^55^ estimated quality-adjusted life year (QALY) disability weights for schistosomiasis (defined in terms of 1 being perfect health and 0 being death) ( Box 1). These were estimated to be 0.9 for moderate-heavy infection and 0.986 for light infection. Despite their similarities, disability weights for QALYs and DALYs are not interchangeable, i.e. one minus a QALY weight does not equal a DALY weight (although some do use this adjustment method) ^120^.

The method used to quantify the effectiveness of interventions and the associated uncertainty needs greater consideration when using schistosomiasis related economic evaluations for informing policy. The choice of metrics will need to reflect the goal and will therefore likely need to be changed as settings move from aiming for morbidity control to interruption of transmission. In most African settings, this will likely mean that the metrics used are currently related to the level of morbidity, whereas in China the metrics will need to reflect the level transmission.

A further source of variation is the time horizon of the economic evaluation ( Box 1 and Table 2). This determines the duration over which the outcomes and costs are calculated and can generate notable differences across studies. Studies with short time horizons may underestimate cost-effectiveness of an intervention.

### Limitations of this analysis

A potential source of bias of the search strategy is that it did not capture economic evaluations published outside of the searched electronic databases, such as grey literature, policy documents/reports, and non-English language publications. In addition, texts without available abstracts or those not clearly identifiable as an economic evaluation may be missed. Efforts were made to minimise this bias by searching the bibliographies of selected studies. There could also be a degree of publication bias, with economic evaluations with negative or less favourable results being less likely to be published. A number of the studies did not have the full text published in English. These were included within the results based on information from their abstracts ( Table 1) but further analysis was limited.

A further limitation was that it was not possible to adjust the results of the studies to a single reference year ^121^. This makes it more difficult to compare the different studies (due to inflation).

## Research needs for future health economic analyses

There are important inconsistencies and research gaps that need to be addressed as we move towards the post-2020 WHO goals. In the following subsections we outline several key research needs and considerations for future economic evaluations ( Box 3 and Box 4).

**Box 3. Key data needs and questions that require additional economic evaluations**

**Key data needs**

- Further cost data on the currently used preventive chemotherapy strategies.
- Data on the cost and effectiveness of alternative strategies (such as WASH, behaviour change, paediatric treatment, snail control and alternative preventive chemotherapy delivery platforms). It will be important that these data are from a range of settings – as the setting may influence the effectiveness of an alternative strategy.
- Data on the link between schistosome infections and morbidity and mortality.
- Further epidemiological data to parametrise the models used for schistosomiasis economic evaluations (such as prevalence and intensity of infection data across all ages, treatment coverage and adherence data, and WASH data).
- Data from hotspot areas.

**Key questions that require additional economic evaluations**

- What is the optimal strategy for achieving and maintaining elimination as a public health problem? This will include investigating whether different preventive chemotherapy strategies and/or complementary interventions (such as snail control) are cost-effective.
- What is the optimal strategy for identifying and controlling hotspots?
- When do alternative treatment strategies (such as test-and-treat) become more cost-effective than currently used preventive chemotherapy strategies?
- Is it more cost-effective to maintain elimination as a public health problem or move towards interruption of transmission?
- What is the cost-effectiveness of different surveillance strategies?

**Box 4. Guidance for future economic evaluations of schistosomiasis interventions**

- Follow standardised guidelines for the reporting of economic evaluations (such as CHEERS ^122^). In particular, clearly state the studies perspective, time horizon and cost year.
- Clearly state how morbidity is modelled and investigate the impact of this on the study’s conclusions. If DALY calculations are used, any changes to the approach used by the Global Burden of Disease study needs to be clearly reported and justified.
- Use economic costs rather than only financial costs (see definitions in Box 1).
- Clearly state the source of the cost data and whether/how the value of donated drugs is included.
- Clearly state the epidemiological setting under investigation (e.g. the pre-control endemicity, schistosome species and age profile of infection). Consider how generalizable the results are to other epidemiological settings.

### Preventive chemotherapy cost data

The costs of preventive chemotherapy vary across different settings ^16– 18^, both within and between countries. A driver is the size of the targeted population ^123– 125^. This is because the delivery costs of preventive chemotherapy can have economies of scale, such that as the number of people treated increases, the cost per treatment tends to decrease ^126, 127^. Another key driver for the costs is the targeted age group. There is very little primary data on the relative cost of school vs community-based preventive chemotherapy ^17^. Many studies in this area make assumptions on the relative cost based on little data. This is an important research gap that needs to be filled to allow further analysis to inform whether and when to switch to community-wide preventive chemotherapy.

It should be noted that preventive chemotherapy delivery costs are not constant and will likely increase over time as countries develop and as expectations regarding the quality of distribution increase. Crucially, the cost per treatment of programmes will also likely increase considerably as they approach their “last mile”, particularly with an end goal of interruption of transmission. This is due to the increase in costs associated with expanding programmes to include harder-to-reach areas and groups ^126, 128^. Hence, a greater understanding of the variation in the costs of preventive chemotherapy across settings and quantifying its impact within subsequent economic evaluations is an important research gap for future studies ^126^.

A notable research gap is the lack of understanding of the costs of integrated NTD control ^129, 130^ and how integration may influence the costs and cost-effectiveness of implementing different control strategies ^126^.

### Evaluation of alternative interventions and strategies

As more data on the costs and effectiveness of interventions become available, economic evaluations of alternative control strategies to preventive chemotherapy need to be conducted, particularly expanding to African settings. This includes WASH ^131^, vaccines ^132^, behaviour change, paediatric treatment, snail control and alternative preventive chemotherapy delivery platforms. Further studies are also needed to quantify the costs and effectiveness of alternative delivery strategies, beyond school-based and community-wide preventive chemotherapy. In particular, the coverage and adherence of different age groups when using various treatment delivery platforms needs to be assessed ^133^. For example, the number of high-risk adults treated through a school-based delivery system and the number of non-enrolled children missed needs to be quantified. It will also be important to investigate the cost-effectiveness of targeting high-risk adults within the community in comparison to targeting the whole community. If a sufficient coverage of high-risk adults could be achieved in addition to school-based treatment, it could be a more cost-effective alternative to mass community-wide preventive chemotherapy. This option is often missed in economics evaluations (due to the lack of data), with most studies only evaluating mass school-based treatment and community-wide preventive chemotherapy.

It is important to consider that as schistosomiasis programmes become more integrated with other NTD programmes or health/education interventions, the costs will likely go down (due to economies of scope ^126^), but the quality of the programme and treatment coverage may also decline.

Importantly, on-going mass treatment may no longer be optimal in low transmission settings and other strategies (such as selective treatment) may need to be considered. Furthermore, the level of treatment coverage that can be maintained with passive treatment at public health facilities needs to be investigated before stopping mass treatment.

### Quantifying the health benefits of interventions

Currently, we would argue that it is difficult to accurately capture the impact of treatment on the morbidity related to schistosomiasis ^99^. There is an urgent need for the development of frameworks that can accurately estimate the number of DALYs averted by schistosomiasis interventions. An ideal framework will need to account for:

i. The differences in how pathogenic different levels of infection are in different age groups (similar to the approach used for STH ^134^). A crucial area of uncertainty for this is the relative burden of light infections ^99^ and how this varies for different species.
ii. Which forms of morbidity are permanent vs reversible with treatment ^99^. This would need to include what morbidity is present in individuals without a current infection ^135^.

Without a better framework, results regarding the benefit of expanding treatment beyond SAC will be highly dependent on assumptions from limited empirical evidence. In particular, overestimating the relative burden of light infections could overestimate the benefit and cost-effectiveness of community-wide treatment (and vice versa) ^99^. Further work is needed to accurately quantify the excess mortality related to schistosomiasis and to reassess the death estimates due to schistosomiasis.

### Epidemiological data, one health and hotspots

Better epidemiological data are needed to parametrise models used for schistosomiasis economic evaluations. This includes prevalence and intensity of infection data across all ages to inform pre-control age profiles of infection, treatment coverage and adherence data, and WASH data. By collecting this data, economic evaluations can be more accurate and informative.

Animal populations have also been shown to be infected with schistosomiasis. The impact of zoonotic transmission to human infection differs by species and this influences the impact of different interventions. Some studies investigating *S. japonicum* included targeting the zoonotic reservoir ( Table 1) but further work is needed to investigate the costs and benefits of applying a one health approach across a range of settings ^136^.

Despite treatment, there are hotspots where infection remains at persistently high levels ^14, 137– 139^. Hotspots can be caused by various programmatic factors such as poor treatment coverage/adherence, movement of infected individuals and intense water contact. Additionally, it is possible that these may be caused by declining drug efficacy as some individuals remain infected following multiple treatment rounds ^140, 141^. These factors pose threats to the effectiveness of treatment programmes and need to be considered in future economic evaluations. Ignoring these could lead to the long-term cost-effectiveness of interventions being overestimated. Economic evaluations also have a role in informing the optimal strategies for identifying and controlling these hotspots.

### The impact of the goal of the intervention

The goal of a programme is an important consideration when interpreting economic evaluations ^16, 18^. Some areas, particularly in Asia, are aiming to move beyond morbidity control and elimination as a public health problem to interruption of transmission ^142^ ( Box 1). Modelling studies have indicated that breaking transmission may theoretically be possible with mass preventive chemotherapy alone at high coverage and compliance ^99, 143^. Field studies and trials are currently underway to confirm if this is feasible in practice ^144, 145^. Moving towards elimination will likely require more intensive strategies, including treatment in low prevalence settings ^14^. It should be noted that an increase in programmatic costs would be required– at least in the short term. More intensive and expensive interventions may not be cost-effective in the context of morbidity control but could be when breaking transmission. For example, in some settings, annual school-based preventive chemotherapy may be the optimal strategy in terms of controlling schistosomiasis-related morbidity, but other more intensive strategies may be more cost-effective when the goal is the interruption of transmission.

## Conclusions

A wide range of economic evaluations of schistosomiasis interventions have been conducted. Based on the identified studies, annual preventive chemotherapy has generally been found to be cost-effective in moderate to high prevalence settings, thereby supporting the WHO recommendation of less frequent treatment in low prevalence settings. However, the cost-effectiveness of mass preventive chemotherapy varies depending on the setting and it is difficult to generalise across species and regions/countries. There are also a growing number of studies evaluating alternative strategies to school-based preventive chemotherapy. Due to the variation in methodology and epidemiological settings, it was not possible to make definitive policy recommendations based on the identified studies. There are several important research gaps that need to be addressed as we move towards the post-2020 WHO goals ( Box 3). In particular, evaluations of interventions other than mass preventive chemotherapy are needed (especially in low transmission settings). Further work is also needed to develop frameworks that can more accurately quantify the health benefits of different strategies. It is also important that future health economics evaluations accurately account for the underlying epidemiology (such as the variation in the age profile of infection) and for the factors which may be driving persistent hotspots.

## Data availability

### Underlying data

All data underlying the results are available as part of the article and no additional source data are required.

### Extended data

Figshare: Supporting Information - Economic evaluations of human schistosomiasis interventions: a systematic review and identification of associated research needs. https://doi.org/10.6084/m9.figshare.11961342.v1 ^15^

This project contains the following extended data:

- Supporting Infomation - Economic evaluations of human schistosomiasis interventions a systematic review and identification of associated research needs.docx (Word file with study search strategy and PRISMA checklist)

Data are available under the terms of the Creative Commons Attribution 4.0 International license (CC-BY 4.0).

### Reporting guidelines

PRISMA checklist for ‘Economic evaluations of human schistosomiasis interventions: a systematic review and identification of associated research needs’. https://doi.org/10.6084/m9.figshare.11961342.v1 ^15^
