# Supplementary material for: Economic evaluations of human schistosomiasis interventions: a systematic review and identification of associated research needs
Source: Wellcome Open Res. 2020 Aug 7;5:45. Originally published 2020 Mar 13. [Version 2] doi: 10.12688/wellcomeopenres.15754.2 (PMC7308887; doi:10.12688/wellcomeopenres.15754.2)
Supplement: Peer review history

# Peer review: Reviewer response for version 2

**DOI:** 10.21956/wellcomeopenres.17795.r39873

**Published:** 2020-08-10

**Authors:** Marina Antillon

**Affiliations:** 1 Swiss Tropical and Public Health Institute, Basel, Switzerland

The authors have responded to my comments satisfactorily. It was a pleasure to work with you.

Are the rationale for, and objectives of, the Systematic Review clearly stated?

Yes

Is the statistical analysis and its interpretation appropriate?

Not applicable

Are sufficient details of the methods and analysis provided to allow replication by others?

Yes

Are the conclusions drawn adequately supported by the results presented in the review?

Yes

Reviewer Expertise:

Economic evaluations, infectious disease transmission modeling, NTDs.

I confirm that I have read this submission and believe that I have an appropriate level of expertise to confirm that it is of an acceptable scientific standard.

# Peer review: Reviewer response for version 1

**DOI:** 10.21956/wellcomeopenres.17277.r38826

**Published:** 2020-06-15

**Authors:** Marina Antillon

**Affiliations:** 1 Swiss Tropical and Public Health Institute, Basel, Switzerland

I commend the authors for an ambitious undertaking comparing economic evaluations throughout the literature of Schistosomiasis (the costs, the different outcomes measured, the different ways of modeling the outcomes, etc). Clearly, the team was well-versed in the recommendations and practices of economic evaluations in general, and as far as I could tell (I am not too familiar in the schistosomiasis literature) they were able to evaluate critically the extent to which the field has been able to follow the best practices of economic evaluations. 

 I hope future investigators, both within the Schistosomiasis circles and in other circles, read the current paper before designing an economic analysis of strategies so that some of the pitfalls of the past studies are avoided. 

 I have a few very minor suggestions: 

 In Table 1, is the year the year of the study's publication or the year in which the interventions were valuated? Could this be indicated in the caption of the table? 

 Did the authors update ICERs to cost values of a common year (i.e. 2017, or 2018)? Because the currencies and costs of some of these countries vary substantially, it's a bit hard to compare these when the values are not of a common year, so this might be worth pointing out as one more element making the literature hard to compare.

 In a similar vein, could it be possible to indicate somewhere what the time horizons for these studies were? For CEA, a 2003 recommendation from the WHO stated a 10-year horizon as a default and alternative horizons as appropriate. In the experience of this reviewer, the horizon doesn't alter the qualitative insights of an economic evaluation, but the horizon may change the ICER based on the time-placement of costs expended and benefits reaped. To reiterate, I do not think that the authors could possibly fix the discrepancies in the literature caused by different horizons used in the studies, but if any difference in horizons are displayed then the reader can decide how important this factor is in the comparison.

 I don't know exactly how many systematic reviews of economic evaluations or costing evaluations related to schistosomiasis exist, but the paper made almost no references to other reviews. Perhaps almost no such reviews exist. However, I know of one review of the unit costs (rather than the cost-effectiveness) of schistosomiasis: Salari *et al. *(2019) ^1^. Could the authors delineate how their observations of the cost components compare to those found by Salari *et al.*?

 Once again, congratulations on a job well done. Such synthesizing efforts are far from straightforward, but are invaluable to understand how policy should move forward.

Are the rationale for, and objectives of, the Systematic Review clearly stated?

Yes

Is the statistical analysis and its interpretation appropriate?

Not applicable

Are sufficient details of the methods and analysis provided to allow replication by others?

Yes

Are the conclusions drawn adequately supported by the results presented in the review?

Yes

Reviewer Expertise:

Economic evaluations, infectious disease transmission modeling, NTDs.

I confirm that I have read this submission and believe that I have an appropriate level of expertise to confirm that it is of an acceptable scientific standard.

## References

[1] : Cost of interventions to control schistosomiasis: A systematic review of the literature. *PLoS Negl Trop Dis*.14(3) : 10.1371/journal.pntd.0008098 e0008098 10.1371/journal.pntd.0008098 32226008PMC7145200

# Response

**Published:** 2020-07-27

**Authors:** Hugo Turner

**Affiliations:** Oxford University Clinical Research Unit, UK

I commend the authors for an ambitious undertaking comparing economic evaluations throughout the literature of Schistosomiasis (the costs, the different outcomes measured, the different ways of modeling the outcomes, etc). Clearly, the team was well-versed in the recommendations and practices of economic evaluations in general, and as far as I could tell (I am not too familiar in the schistosomiasis literature) they were able to evaluate critically the extent to which the field has been able to follow the best practices of  economic evaluations. 

I hope future investigators, both within the Schistosomiasis circles and in other circles, read the current paper before designing an economic analysis of strategies so that some of the pitfalls of the past studies are avoided. 

I have a few very minor suggestions: 

In Table 1, is the year the year of the study's publication or the year in which the interventions were valuated? Could this be indicated in the caption of the table? 

**Response: The year references to the year of publication. We adapted the table to make this clearer**

Did the authors update ICERs to cost values of a common year (i.e. 2017, or 2018)? Because the currencies and costs of some of these countries vary substantially, it's a bit hard to compare these when the values are not of a common year, so this might be worth pointing out as one more element making the literature hard to compare.

**Response:   We did not adjust for inflation to a common year. This would have been ideal however was not possible because several of the studies did not report clearly the original cost year and exchange rates. We have made this point clearer within the text.**

***Methods:***

***….***

***The studies estimates were kept in their original cost year and not adjusted to a single reference year.***

***Limitations:***

***…..***

***A further limitation was that it was not possible to adjust the results of the studies to a single reference year. This makes it more difficult to compare the different studies.***

In a similar vein, could it be possible to indicate somewhere what the time horizons for these studies were? For CEA, a 2003 recommendation from the WHO stated a 10-year horizon as a default and alternative horizons as appropriate. In the experience of this reviewer, the horizon doesn't alter the qualitative insights of an economic evaluation, but the horizon may change the ICER based on the time-placement of costs expended and benefits reaped. To reiterate, I do not think that the authors could possibly fix the discrepancies in the literature caused by different horizons used in the studies, but if any difference in horizons are displayed then the reader can decide how important this factor is in the comparison.

**Response: This is a very good point. We have added the reported time horizon for the studies in Table 2 and added the following into the text within the discussion.**

***“Variation in the methods used to quantify the effectiveness of interventions***

***….***

***“A further source of variation is the time horizon of the economic evaluation (Box 1 and Table 2). This determines the duration over which the outcomes and costs are calculated and can generate notable differences across studies. Studies with short time horizons may underestimate cost-effectiveness of an intervention.”***

I don't know exactly how many systematic reviews of economic evaluations or costing evaluations related to schistosomiasis exist, but the paper made almost no references to other reviews. Perhaps almost no such reviews exist. However, I know of one review of the unit costs (rather than the cost-effectiveness) of schistosomiasis: Salari *et al. *(2019) ^1^. Could the authors delineate how their observations of the cost components compare to those found by Salari *et al.*?

**Response:  At the time we submitted there were no other relevant reviewers regarding economic evaluations or costing evaluations related to schistosomiasis to existed. We have now added the reference to the Salari *et al. *(2019) paper.**

***“The average delivery cost of annual preventive chemotherapy using praziquantel was typically assumed (or in some cases estimated) to be between US$0.20-0.50 per treatment [50]. This is consistent with a recent systematic review by Salari et al [ref A1], which found that the average delivery cost of preventive chemotherapy for schistosomiasis (with or without an educational component) was US$0.30 per treatment. However, the methods used to parameterise the costs of different interventions within these economic evaluations varied widely making it difficult to understand the relative costs of different interventions and how costs may vary across countries/regions. This variation also made it difficult to directly compare the different studies. Importantly, Salari et al. [ref A1] also found that the degree of transparency of most of the costing studies of schistosomiasis interventions they identified was limited. An important consideration is whether financial or economic costs are being used (Box 1). Economic costs are considered the gold standard within economic evaluations as they better reflect the sustainability and replicability of interventions. Many studies did not formally state if they were using financial or economic costs. There was also variation regarding whether the cost of donated praziquantel had been included within the analysis as an economic cost (Table 2).”***

Once again, congratulations on a job well done. Such synthesizing efforts are far from straightforward, but are invaluable to understand how policy should move forward.

# Peer review: Reviewer response for version 1

**DOI:** 10.21956/wellcomeopenres.17277.r38175

**Published:** 2020-03-31

**Authors:** William Evan Secor

**Affiliations:** 1 Division of Parasitic Diseases and Malaria, Centers for Disease Control, Atlanta, GA, 30329, USA

This manuscript provides a strong background for understanding economic analyses related to schistosomiasis. For those with little background in this area, the definitions and examples provided in the boxes are very helpful. For example, the distinction between cost-effectiveness and cost-benefit. 

 Although the paper is extremely well written, the authors were not able to accomplish their original intent to systematically review the available data and draw relevant conclusions. This was because the different existing studies lacked standardization to the extent that they simply were not comparable. To that end, I would encourage the authors to add 2 more boxes to the manuscript. The first one could be a listing of what, in their opinion, are the additional economic evaluations that are needed to inform policy and guideline development. Is there, for example, a need to show at what prevalence switching from MDA to test-and-treat would be a better option? Also, would it be useful to have data on mollusciciding costs and benefits or effects in areas endemic for schistosome species that are not typically zoonotic? Knowing what research is critically needed can help direct investigators to developing more relevant studies.

 Secondly, and related, a box containing the key information that should be collected in any future studies would be helpful to avoid the pitfalls of variations in methodology that precluded this study from drawing generalizable conclusions. The authors mention some of these key elements in the text (e.g., collecting financial costs rather than just economic ones, defining the pre-control endemnicity, noting the age profile of infection) but having something of a checklist would be helpful to any future investigators in this area. This paper is really a nice starting point for researchers considering conducting economic evaluations, it would be an even better contribution to the literature if it could provide guidance on how best to conduct those studies that will make future systematic reviews able to make policy recommendations.

Are the rationale for, and objectives of, the Systematic Review clearly stated?

Yes

Is the statistical analysis and its interpretation appropriate?

Not applicable

Are sufficient details of the methods and analysis provided to allow replication by others?

Yes

Are the conclusions drawn adequately supported by the results presented in the review?

Yes

Reviewer Expertise:

Immunology, diagnostics, control evaluations of schistosomiasis.

I confirm that I have read this submission and believe that I have an appropriate level of expertise to confirm that it is of an acceptable scientific standard.

# Response

**Published:** 2020-07-27

**Authors:** Hugo Turner

**Affiliations:** Oxford University Clinical Research Unit, UK

Response: These are very good points. We have added the following boxes (box 3 and box 4) to add in these points.

**Box 3: Key data needs and questions that require additional economic evaluations.**

**Key data needs**

- Further cost data on the currently used preventive chemotherapy strategies.
- Data on the cost and effectiveness of alternative strategies (such as WASH, behaviour change, paediatric treatment, snail control and alternative preventive chemotherapy delivery platforms). It will be important that these data are from a range of settings – as the setting may influence the effectiveness of an alternative strategy.
- Data on the link between schistosome infections and morbidity and mortality.
- Further epidemiological data to parametrise the models used for schistosomiasis economic evaluations (such as prevalence and intensity of infection data across all ages, treatment coverage and adherence data, and WASH data).
- Data from hotspot areas.

 
**Key questions that require additional economic evaluations**

- What is the optimal strategy for achieving and maintaining elimination as a public health problem? This will include investigating whether different preventive chemotherapy strategies and/or complementary interventions (such as snail control) are cost-effective.
- What is the optimal strategy for identifying and controlling hotspots?
- When do alternative treatment strategies (such as test-and-treat) become more cost-effective than currently used preventive chemotherapy strategies?
- Is it more cost-effective to maintain elimination as a public health problem or move towards interruption of transmission?
- What is the cost-effectiveness of different surveillance strategies?

**Box 4: Guidance for future economic evaluations of** **schistosomiasis interventions. ** 

- Follow standardised guidelines for the reporting of economic evaluations (such as CHEERS [ref A1]). In particular, clearly state the studies perspective, time horizon and cost year.
- Clearly state how morbidity is modelled and investigate the impact of this on the study’s conclusions. If DALY calculations are used, any changes to the approach used by the Global Burden of Disease study needs to be clearly reported and justified.
- Use economic costs rather than only financial costs (see definitions in Box 1).
- Clearly state the source of the cost data and whether/how the value of donated drugs is included.
- Clearly state the epidemiological setting under investigation (e.g. the pre-control endemicity, schistosome species and age profile of infection). Consider how generalizable the results are to other epidemiological settings.
